# System identification theory in myocardial perfusion modeling of dynamic contrast-enhanced MRI

**DOI:** 10.1186/1532-429X-11-S1-P277

**Published:** 2009-01-28

**Authors:** Jean-Paul Vallée, Marko Ivancevic, Jean-Luc Daire, Dominique Didier, Michel Kocher

**Affiliations:** Radiology/Geneva University Hospital, Geneva-14, Switzerland

**Keywords:** Myocardial Perfusion, Myocardium Blood Flow, Injection Rate, Arterial Input Function, System Identification Method

## Introduction

Tissue perfusion and blood volume can be quantified by MRI after contrast media (CM) injection and a one compartment model that yields K1, the blood to tissue transfer constant (related to myocardial blood flow MBF) and Vd, the CM distribution volume. Using system identification theory and computer simulations, the confounding effects of volume and rate of a contrast injection on the reliability of K1 estimation was recently evaluated (Aerts MRM 2008; 59:1111). The simulations predict that the K1 is most reliable with a high injection volume administered in a single injection, where high rates only modestly improve K1. However, the data used for the simulation were taken from a model of rectal cancer. It is not known how these simulations apply to myocardial perfusion at rest and at stress. Therefore, the aim of this study was to apply system identification theory on realistic data encountered in cardiac patients.

## Methods

**Figure 1:**
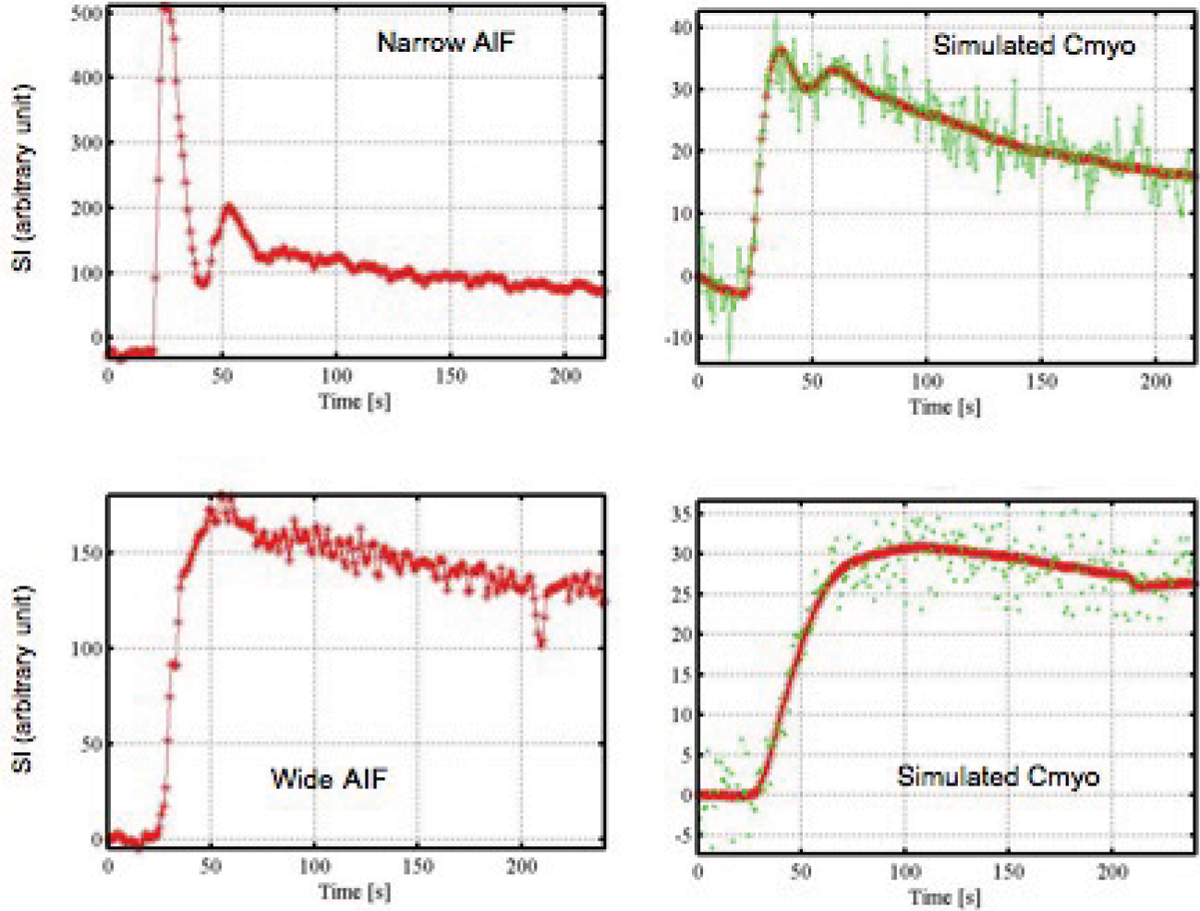
Figure 1

To evaluate the effect of the bolus shape on the model, two arterial input functions (AIF), were derived from real data (narrow AIF with a small CM volume and fast injection rate 0.035 mmol/kg at 5 cc/sec and wide AIF with a larger CM volume and slower injection rate 0.08 mmol/kg at 0.5 cc/sec as shown in Figure 1) and used as input stimuli. Using constant rest and stress values for K1 (0.6 ml/ming/g and 2.4 ml/min/g) and Vd (15%), time transit curves (Cmyo) have been simulated by the discrete transfer function of the one compartment model derived from the Laplace transform (see Figure 1). The output error (OE) method was used as a system identification method to estimate K1 and Vd. Finally, estimated values of K1 and K2 are described for different realistic noise indices (the standard deviation of a zero mean Gaussian process varies from 0 to 10%) and different under-sampling levels (from 1 to 4×). Bias (fitted value/true value) in % and error (std dev/mean) in % of fitted K1 and Vd as well as the Bode diagram were obtained for each cases.

## Results

At rest, no significant difference in bias and standard deviation were observed with the identification process between the two shapes of the arterial input function (narrow and wide). Under-sampled curves introduce an increase of standard deviation of rest K1 and K2 but with no significant effect on the bias (< 1% for noise up to 5%). At stress (see Figure 1), there was no significant bias for both arterial input functions red and green curves of Figure 1) as well as for the wide undersampled function (black curve). However, almost 5× increase of bias was observed in the undersampled narrow function (blue curve). The standard deviation was increased by undersampling as well by a slow injection rate. Regarding the distribution volume Vd, accurate measurement was obtained for all the conditions (bias < 1% for a noise of 0.05 and standard deviation < 4%).

**Figure 2:**
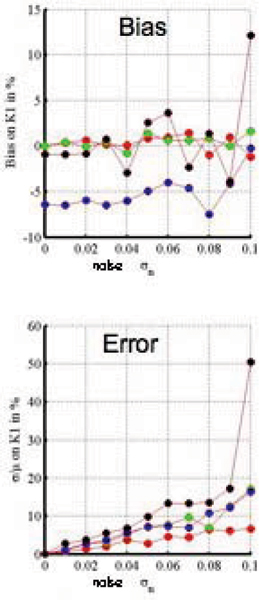
Figure 2

The Bode diagram showed a similar pattern around the cut-off frequency for the narrow and wide AIF at rest as well as for the wide AIF at stress but not for the narrow undersampled AIF explaining the lowest performance of this AIF. See also Figure 2.

## Conclusion

At rest, both slow and fast injection rates as well as undersampling up to 1 image/4 seconds yield accurate measurements of the myocardium blood flow and distribution volume. At stress the myocardium blood flow measurement is less accurate in case of a fast injection of a small volume of CM. The simulations indicated that MBF measurements at stress can improved by slowing the injection rate for the same sampling frequency.

